# Grasps Recognition and Evaluation of Stroke Patients for Supporting Rehabilitation Therapy

**DOI:** 10.1155/2014/318016

**Published:** 2014-09-02

**Authors:** Beatriz Leon, Angelo Basteris, Francesco Infarinato, Patrizio Sale, Sharon Nijenhuis, Gerdienke Prange, Farshid Amirabdollahian

**Affiliations:** ^1^Adaptive Systems Research Group at the School of Computer Science, University of Hertfordshire, Hatfield, Hertfordshire AL10 9AB, UK; ^2^IRCCS San Raffaele Pisana, Via di Val Cannuta 247, 00166 Roma, Italy; ^3^Roessingh Research and Development, Roessinghsbleekweg 33b, 7522 AH Enschede, The Netherlands

## Abstract

Stroke survivors often suffer impairments on their wrist and hand. Robot-mediated rehabilitation techniques have been proposed as a way to enhance conventional therapy, based on intensive repeated movements. Amongst the set of activities of daily living, grasping is one of the most recurrent. Our aim is to incorporate the detection of grasps in the machine-mediated rehabilitation framework so that they can be incorporated into interactive therapeutic games. In this study, we developed and tested a method based on support vector machines for recognizing various grasp postures wearing a passive exoskeleton for hand and wrist rehabilitation after stroke. The experiment was conducted with ten healthy subjects and eight stroke patients performing the grasping gestures. The method was tested in terms of accuracy and robustness with respect to intersubjects' variability and differences between different grasps. Our results show reliable recognition while also indicating that the recognition accuracy can be used to assess the patients' ability to consistently repeat the gestures. Additionally, a grasp quality measure was proposed to measure the capabilities of the stroke patients to perform grasp postures in a similar way than healthy people. These two measures can be potentially used as complementary measures to other upper limb motion tests.

## 1. Introduction

Stroke survivors are often unable to perform the fine motor control activities which are required during activities of daily living, amongst which grasping is one of the most recurrent. Robots represent an appealing tool for exercise-based approach in neurorehabilitation due to their ability to deliver repetitive training.

Previous studies indicate that the inclusion of robot rehabilitation training improves short- and long-term motor control of the impaired upper limb of patients after a stroke [1, 2]. However, evidence of the transfer of robotic training effects to activities in daily life is limited [3]. Therefore, the inclusion of functional tasks, such as grasping objects, is vital to increase the practice and improvement in such activities for stroke rehabilitation [4, 5]. A next step in this direction is to incorporate the detection of various grasps in the robot rehabilitation frameworks and to evaluate their quality in order to detect any improvement in the rehabilitation process.

**Figure 1:**
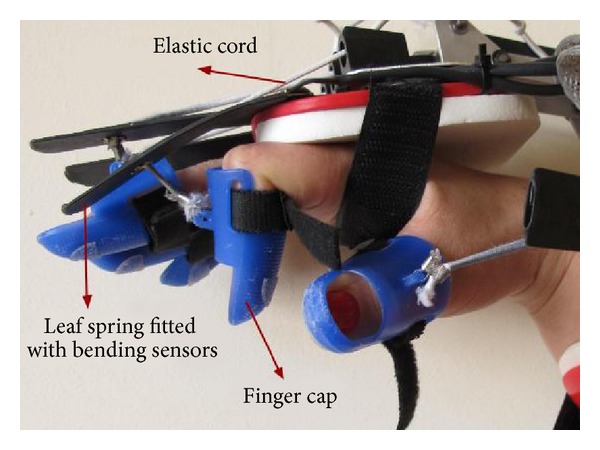
SCRIPT passive orthosis showing the bending sensors and leaf springs.

The SCRIPT (Supervised Care & Rehabilitation Involving Personal Telerobotics) project aims at delivering an affordable system for home-based rehabilitation of the hand and wrist for stroke survivors [6]. A passive exoskeleton (Figure 1) has been developed within the project in order to facilitate the patients' fingers and wrist extension. Self-administered training at home is performed through repetitive interaction with games based on functional exercises (rehabilitation games) to enhance engagement. The games are controlled by wearing the orthosis and performing several arm and hand movements. The challenge is to use the limited set of sensors provided by the device to recognize the various grasp postures and incorporate them into the games.

In previous work [7], we implemented and tested different methods for recognizing four different grasp postures performed while wearing the SCRIPT orthosis. We found that with the support vector machines (SVM) method, we could achieve an overall accuracy of more than 90% with small computational time. SVM has been successfully applied to the classification of a variety of biomedical conditions [8–10] and more specifically to study different aspects of stroke patients, including the classification of carotid artery plaques [11], the study of dietary patterns [12], or using readings of a shoe-based sensor to identify sitting, standing, and walking postures [13]. However, few studies have been reported on the classification of grasp postures. Tavakolan et al. [14] used SVM for pattern recognition of surface electromyography signals of four forearm muscles in order to classify eight hand gestures. On the other hand, Puthenveettil et al. [15] used linear discriminant analysis to classify hand preshapes in poststroke patients using data from the CyberGlove. In this study, the SVM approach was selected to perform the gesture recognition.

We conducted an investigation in a group of eight poststroke patients to determine the accuracy of the gesture recognition wearing the SCRIPT device using the SVM approach and compared it with the samples obtained for a group of ten healthy subjects, and their relationship to the level of hand impairment. The goal was to assess the grasp recognition with this particular user group, in order to confirm its suitability prior to deployment in a larger scale clinical evaluation of the prototype orthotic device and the SCRIPT system. Successful grasp recognition will provide a more versatile set of gestures to the therapeutic human-machine interaction system that acts as a medium to support home-based rehabilitation. After a given grasp posture is recognized, the aim is to evaluate the quality of the grasp in order to measure the patient progress throughout the rehabilitation process. In this study, we propose a grasp quality measure that can be calculated with the available sensor readings of the orthosis. These measures are compared with the results of other commonly used upper limb motion tests such as the action research arm test (ARAT) [16].

## 2. Materials and Methods

### 2.1. Device

The SCRIPT passive exoskeleton [17] is a passive device which applies external extension torques on the fingers through five leaf springs which are connected to the finger caps through an elastic cord (Figure 1). The elastic cord enables the fingers freedom of movement relative to the leaf spring and also allows adjusting the level of extension support provided by the device. The leaf springs are fitted with commercial resistive flex sensors [18] which measure their flexion with a resolution of 1 degree. However, the deflection of the leaf spring is not the actual flexion angle of the finger but the two quantities are related by a monotonically increasing function [17]. Subjects are free to laterally abduct/adduct the fingers and oppose the thumb, but these movements are not supported nor sensed by the orthosis. The orthosis measures only overall finger flexion in a range from 0 to 90 degrees and wrist flexion and extension in a range from 90 to −45 degrees.

It is important to note that this passive orthosis, as many of those in the field of rehabilitation robotics, may not conform to the conventional definition of a robot, although consisting of sensors, passive actuators, and a decision making component. However, our research is applicable in the field of robot-assisted training and rehabilitation robotics, where sensors are utilized towards benchmarking motor performance aiding a meaningful two-way interaction between the human and the machine.

### 2.2. Study Participants

Two groups of participants were recruited for this study: a group of healthy subjects and a group of stroke patients. In the first group, ten healthy subjects with no previous injuries of fingers, hand, or wrist volunteered to participate in this study (Table 1). This study was carried out at the University of Hertfordshire, UK, approved by the university ethics committee (Ethics protocol number COM/ST/UH/00008) and at the IRCCS San Raffaele Pisana (Rome, Italy). Participants were recruited among faculty staff by advertising on an internal mailing list.

The group of stroke patients were selected with the criteria used in the SCRIPT project in order to enable them the use of the orthosis [19]. They were patients with a unilateral ischemic or hemorrhagic stroke between 6 months and 5 years ago. They had limitations in arm and hand function, while being able to actively flex the elbow by at least 15° and to actively flex their fingers by a quarter of their passive range. They also had the ability to understand and follow instructions. For stroke patients, individually fitted orthoses were used. With these criteria, a total of eight patients were selected (Table 2). One patient was recruited at the Rehabilitation Centre Het Roessingh (Enschede, the Netherlands) and seven at the IRCCS San Raffaele Pisana (Rome, Italy). In both cases, the experiment was part of an on-going clinical study, for which ethical approval had been obtained at the respective local ethics committees.

The action research arm test (ARAT) [16] and Fugl-Meyer assessment (FM) [20, 21] have been used as quantitative measures to evaluate the arm motor recovery after stroke. The ARAT consists of four sections: (A) grasp, (B) grip, (C) pinch, and (D) gross arm movement. The FM assessment for the upper extremity consists on a scale of 66 points divided in three subsections: proximal (shoulder and elbow), distal (wrist and hand), and coordination (test of tremor, dysmetria, and time). Details of the results of these tests for the stroke patients can also be seen in Table 2.

### 2.3. Grasp Gestures

**Figure 2:**
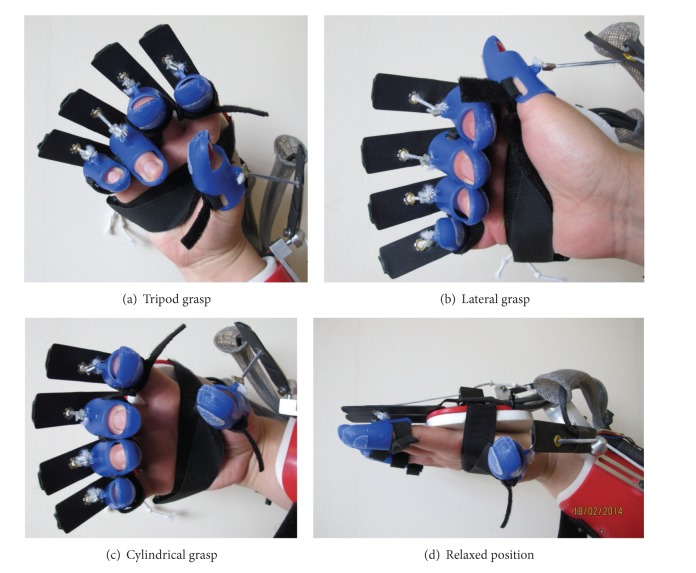
Selected gestures to be recognized performed wearing the SCRIPT device.

In recent literature, several activities of daily living have been identified as the most important to train after stroke [4, 22, 23]. They include eating with cutlery, drinking, holding objects while walking, taking money from purse, open/close clothing, combing hair, and knob manipulation. We have selected, in agreement with healthcare professionals, three grasp gestures needed in order to perform these tasks (shown in Figure 2). Two are classified as precision grips: the tripod (the thumb opposes the index and middle finger) and the lateral grasp (the thumb holds an object against the side of the index finger). The third one, the cylindrical grasp, is classified as a power grasp (all fingers make contact with the object). Keller et al. [24] identified the tripod and the lateral grasps as the most frequently used prehensile patterns for static and dynamic grasping, respectively. The relaxed posture of the hand was used as the forth gesture in order to enable the recognition when the subjects were not performing any grasp.

Two of the selected gestures are evaluated in the ARAT: the tripod grasp is tested in section A (grasp subtest) and the cylindrical grasp in section B (grip subtest) of ARAT. It is expected that the recognition of the gestures is related to the ability to move the hand measured by ARAT.

### 2.4. Grasp Recognition Method

The problem of hand posture detection has been previously approached using vision-based [25, 26] or glove-based [25, 27–29] methods depending on the constraints of the specific applications. In our case, given the bulk of the device, vision-based approaches for the recognition of the hand postures are not suitable as the hand is practically occluded and therefore the recognition should be based on the sensory readings for each finger provided by the exoskeleton sensors. In a previous work, we compared several glove-based approaches to recognize grasp postures performing experiments on five healthy participants wearing the SCRIPT passive exoskeleton [7]. We compared three methods: one based on the statistics of the flexion data, another based on neural networks, and finally one based on support vector machines (SVM). We found that with the last method, we could achieve an overall accuracy of more than 90% with small computational time (<60 ms). Therefore, in this study, we used the SVM approach to determine the accuracy of recognition for healthy participants and stroke patients wearing the SCRIPT device.

SVM is a popular machine learning technique for classification. A support vector machine is a supervised learning classifier that constructs a set of hyperplanes in a high-dimensional space (support vectors) that are used to classify the data. A good separation is achieved by the hyperplane that has the largest distance to the nearest training data point of any class. The hyperplanes are found solving the following optimization problem [30]:

(1) min⁡ω,b,ε⁡12ωTω+C∑i=1lεisubject to yi(ωTφ(xi)+b≥1−εi), εi≥0,

where {(*x*_*i*_, *y*_*i*_)∣*x*_*i*_ ∈ *R* ^*p*^, *y*_*i*_ ∈ −1,1} are the training set of *l* instance-label pairs, *x*_*i*_ is *p*-dimensional real vector, *ω* the normal vector of the hyperplane, and *C* > 0 the penalty parameter of the error term. The training vectors *x*_*i*_ are mapped into a *p*-dimensional spaces by the function *φ* and in order to create nonlinear classifiersa kernel function is used. In our work, we used a radial basis function (RBF) as the kernel function, given that it can handle the case when the relation between class labels and attributes is nonlinear [31]. It is defined as

(2) $$\begin{array}{c} K\left(x_{i}{,x}_{j}\right)=\exp\left(-\gamma {||x_{i}{-x}_{j}||}^{2}\right),\;\gamma >0, \end{array}$$

where *γ* is the kernel parameter. Therefore, two parameters are needed: *C*and *γ*. In order to find the best values for these parameters, we used a *v-fold* cross-validation technique, dividing the training set for one subject into *v* subsets of equal size and testing the classifier on the remaining *v* − 1 subsets. In this work, *v*  was taken as 5, the value of the cost parameter *C* was varied as *C* = 2^*x*^, *x* = {−5,…, 5} and the value of the kernel parameter *γ* was varied* as γ* = 2^*y*^, *y* = {−4,…, 0}. The values that gave the highest validation accuracy were: *C* = 4 and *γ* = 1.

The method was implemented in Python using the* LIBSVM* package (http://www.csie.ntu.edu.tw/~cjlin/libsvm). The flexion angles were normalized in the range from 0 to 1 (corresponding to 0 to 90 degrees) and the selected error to stop the training phase was set to 0.001.

### 2.5. Grasp Evaluation Method

In the field of robotics, many grasp quality measures have been developed that allow the comparison of different aspects of the robotic grasp [32]. In [33], the most common robot grasp quality measures have been adapted to the evaluation of the grasp of the human hand. From this set of quality measures, the one proposed by [34], which measures how close a given grasp is to a reference posture, is the only one that can be used with the sensor information provided by the SCRIPT orthosis. This index has been adapted for this study as follows:

(3) $$\begin{array}{c} GQ=1-\overset{n}{\underset{i=1}{\sum }}\omega_{i}{\left(\frac{y_{i}-a_{i}}{R_{i}}\right)}^{2}, \end{array}$$

where *n* is the number of hand joints, *ω*_*i*_ is a weight factor, *y*_*i*_ is the current finger flexion angle, and *R*_*i*_ is the joint angle range used to normalize the index calculated as the maximum between the reference posture *a*_*i*_ and either the upper or lower angle limit. The index has to be maximized, so that the grasp is optimal when all joints are at the reference posture, having a quality measure of one, and it goes to zero when all its joints are at their maximum angle limits.

The reference posture *a*_*i*_ is taken in this case as the one performed by the healthy subjects. This measure then enables the evaluation of how far is a poststroke patient from performing a grasp in a way similar to a healthy subject. However, the postures obtained from healthy subjects performing each gesture are likely to have variations between subjects, especially when some of the fingers were not playing an active role in the grasp (e.g., the ring and little fingers in the tripod grasp). In order to consider this variance, we have included a weight factor *ω*_*i*_ that will give high scores to fingers whose postures have small standard deviation and vice versa. The corresponding weights for each finger and a given gesture can be calculated as

(4) $$\begin{array}{c} w_{ig}=\frac{\alpha_{g}}{\sigma_{ig}}, \end{array}$$

where *i* and *g* are the given finger and gesture; *σ*_*ig*_ is the standard deviation of the finger flexion over all subjects for the given finger and grasp; and *α*_*g*_ is calculated as

(5) αg=5∏i=15σig×((∏σig)i=1,2,3,4+(∏σig)i=1,3,4,5  +(∏σig)i=1,2,4,5+(∏σig)i=1,2,3,5  +(∏σig)i=2,3,4,5)−1.

### 2.6. Experimental Protocol

The participants took part in one session lasting half an hour conducted by a researcher or a therapist. They were asked to wear a SCRIPT passive orthosis on the impaired hand or one of their hands (in the case of healthy participants) while sitting in front of a PC. Subsequently, they were instructed to mimic the picture of a gesture shown on the screen (Figure 2). The participant then confirmed that he/she achieved the desired gesture and then, pressing a button on the screen, the flexion angles of the gesture were saved. After confirmation, they were asked to relax the hand and press a button. At that moment, the angles of the relaxed posture were also saved.

Each subject performed 6 repetitions of each gesture in a pseudorandom sequence, resulting in the capture of 24 gestures. Data were then postprocessed by Python ad hoc applications. The results of the classification system were calculated using the following values for each gesture *i*:

- True positives (TP): gestures correctly classified as gesture *i*
- False negatives (FN): gestures *i* incorrectly classified as other gestures
- False positives (FP): other gestures incorrectly classified as gesture *i*
- True negatives (TN): other gestures correctly classified as nongesture *i*.

These are commonly used to evaluate the sensitivity and specificity of a clinical test in its ability to confirm or refute the presence of a disease. The sensitivity (true positive rate) refers to the ability of the test to correctly identify those patients with the disease and the specificity (true negative rate) refers to the ability to correctly identify those patients without the disease [35]. In this study, the outcome of the test is not binary therefore we calculated the performance measures focusing on each gesture. We used the accuracy (ability to correctly identify positive and negatives), true positive rate (ability to correctly identify the positives), and false positive rate (lack of ability to correctly classify the negatives). The last one is measuring the opposite of the specificity as it enables us to focus on evaluating the recognition performance for a given gesture instead of looking at the classification of other gestures. They are calculated as follows:

(6) $$\begin{array}{c} \text{Accuracy}=\frac{\text{TP}+\text{TN}}{\text{Total}\;\text{testing}\;\text{data}},\\ \text{True}\;\text{positive}\;\text{rate}\;\left(\text{TPR}\right)=\frac{\text{TP}}{\text{TP}+\text{FN}},\\ \text{False}\;\text{positive}\;\text{rate}\;\left(\text{FPR}\right)=\frac{\text{FP}}{\text{TN}+\text{FP}}. \end{array}$$

### 2.7. Data Analysis

The data acquired for each subject was divided into two sets for training and testing purposes. As the patients will need to perform the training procedure each time before starting the games (or the games will not be able to reliably recognize their gestures), the less number of samples required to train the model the better. In [7] it has been shown that a high accuracy can be achieved with 4 training samples, thus allowing very short calibration time and making this approach suitable for home-based rehabilitation. Then a training set using 4 samples per gesture was used to train the model. Results were considered taking into account all the possible permutations of 4 training samples.

The overall results of the recognition performance are summarized as median and interquartile range (using box plots) differentiated between the participant's type (healthy or stroke patient). The information provided by the different performance measures is compared using a Pearson correlation in order to determine if we can rely on one measurement for the recognition assessment. We also compared the variability of the flexion angles over all subjects performing the different grasp gestures and the results of the accuracy of gesture recognition per participant.

Additionally, the results of the proposed grasp quality measure are also presented summarized using box plots showing the variation between the participant's type, the different gestures and the intervariability between the participants. In order to assess how these three parameters influenced the results of the grasp quality measure, a multiple regression analysis was performed. This type of analysis can be used to consider multiple independent variables calculating the least square estimates for a data set [36]. Using this analysis, we can devise our model using the following equation:

(7) GQi=b0+b1 Participant  typei+b2 Grasp  typei+b3 Subjecti+εi,

where *b*_*o*_ represents the constant and *ε* is the modelling error. In this case, predictors are classification variables with more than two categories, therefore dummy coding is required to include them in the regression equation. The technique to do this coding is to create an independent variable for each independent category except one as a dichotomy. The omitted variable provides a baseline for comparison while avoiding multicollinearity [36]. In this analysis, we used “healthy participants,” “relaxed posture,” and “subject 1 (healthy)” as our base line for each one of our predictors. The “Enter” method was used in order to force the model to consider all variables as significant variables in the model.

It is intuitive to assume that the level of impairment of the patients should be correlated with their ability to consistently perform the gestures in a similar way (recognition accuracy) and similar to the ones performed by healthy subjects (GQ measure). As the ARAT and FM tests are common ways to evaluate the level of capabilities of the upper limb, we correlated the accuracies of recognition and the grasp quality with the results of these test using the Pearson coefficient. The IBM SPSS statistical package for Windows version 21.0. was used to perform the analysis of the data.

## 3. Results

This section presents the results of the experiments in two parts: the results of the recognition of the different gestures performed by healthy subjects and stroke patients and the results of the evaluation of those grasps.

### 3.1. Grasp Recognition Results

The results of the true positive rate of the training and testing phases of the experiments are presented in Table 3. The number of gestures correctly recognized greatly increased from the training to the testing phase. It can clearly be seen that the testing overall recognition performance of gestures performed by the stroke patients is lower than the ones performed by the healthy subjects, as it was expected, but still they produced a high percentage of true positive recognized gestures (TPR mean of 75%). The computational time taken for training the model was on average 1.98 ms for healthy subjects and 2.21 ms for stroke patients.

**Figure 3:**
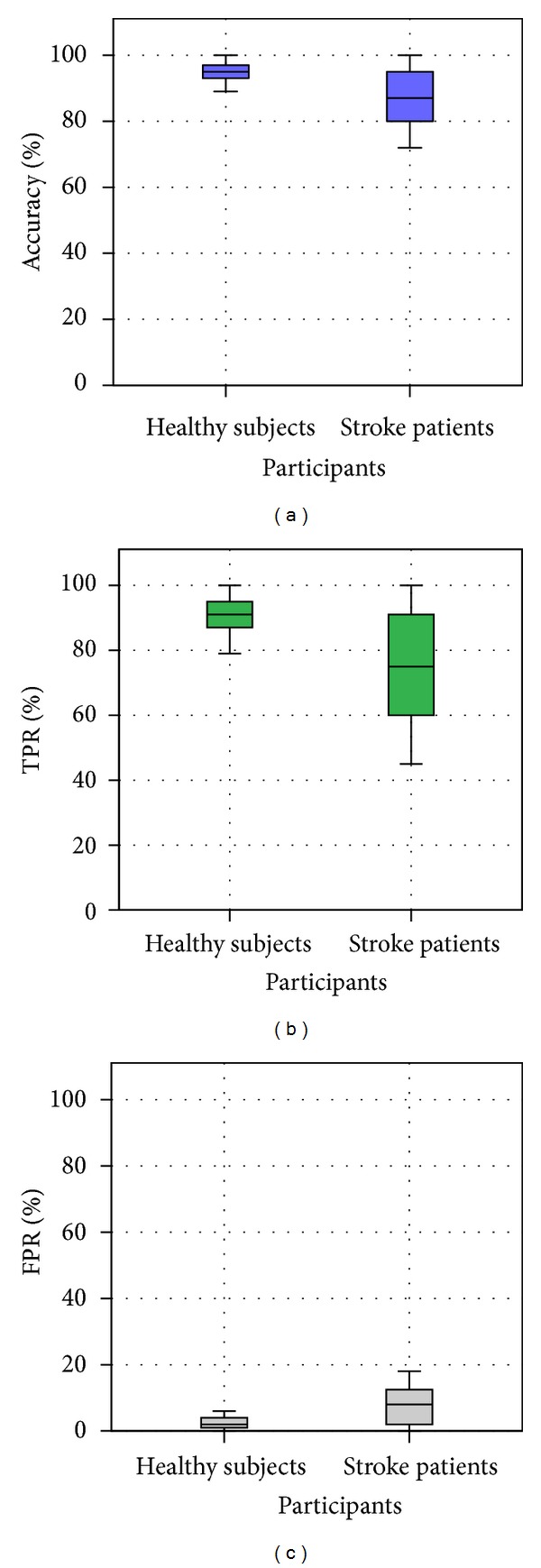
Performance measures evaluating the recognition of grasp postures: (a) accuracy, (b) true positive rate and, (c) false positive rate. Results are presented discriminated between healthy and stroke participants.

In order to evaluate in more detail the recognition results, the selected performance measures are shown in Figure 3. Ideally, the accuracy and true positive rate should be close to 100% and the false positive rate close to zero. In this case, the median accuracy of recognition of gestures performed by healthy subjects was over 95% and 87% for stroke patients. The true positive rate showed lower values with respect to the accuracy: median values for healthy subjects of over 91% and for stroke patients of over 75%. The healthy subjects showed median values of false positive rates of 2% and 8% for stroke patients.

**Figure 4:**
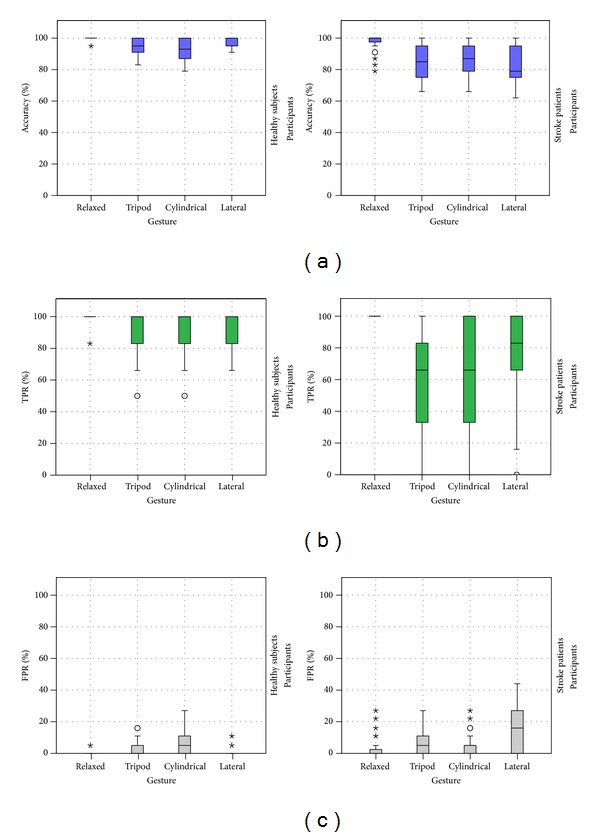
Performance measures for each gesture evaluating the recognition of hand postures: (a) accuracy, (b) true positive rate, and (c) false positive rate.

The different performance measures can also provide information about the specific recognition of each gesture. Specifically, the true positive rate refers to the ability of the method to correctly identify a specific gesture, whilst the false positive rate refers to the percentage of gestures wrongly classified.  Figure 4 shows the accuracy, true positive rate, and false positive rate for each gesture. The difficulty of recognition of the different grasp gestures is different for the healthy subjects or stroke patients. The relax posture is a clearly distinctive gesture, therefore showing the best accuracies, best TPR and FPR values close to zero. For healthy subjects, the tripod and cylindrical gestures were the most difficult to be recognized and the most misrecognized. For stroke patients, the three gestures presented similar difficulties to be recognized and the tripod and lateral grasp were the most misrecognized.

Table 4 presents the Pearson correlation values of the different performance measures for the given conditions. The accuracy is inversely correlated with the false negative rate over all conditions (correlation coefficient C < −0.97 with statistical significance) and also highly correlated with the TPR (C > 0.99). The different measures of performance provide specific information on the performance of recognition, but the accuracy could be selected as the overall measure of recognition performance. Therefore, for the following analysis, the accuracy will be used.

It is expected that the grasping capabilities of the participants affect the recognition of hand postures, especially in the case of stroke patients. Therefore, we also studied the variability of the finger flexion to produce the different gestures and their impact on the recognition.

**Figure 5:**
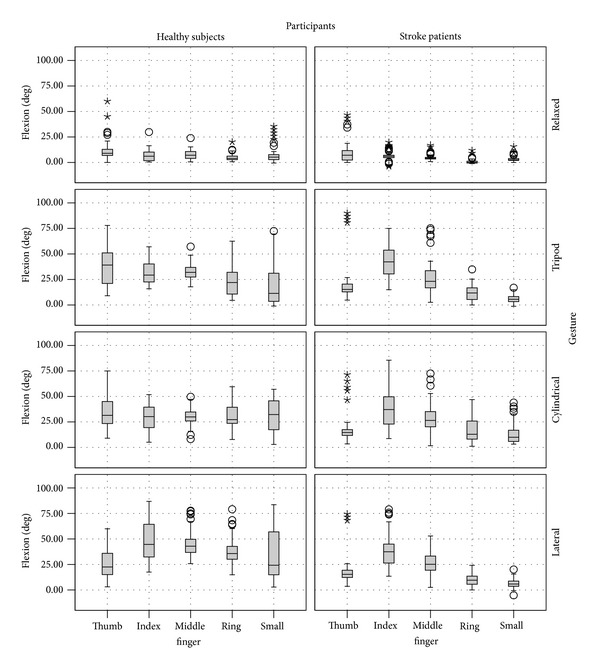
Variability of the different finger flexion values produced by all subjects while performing the various gestures.

Figure 5 shows a summary of the variability of the flexion angles over all subjects performing the different grasp gestures. As expected, the relaxed posture is the most consistent over all gestures. For healthy subjects, the ring finger and little finger are the ones with greater variation as they can freely move and are not actively participating in the tripod grasp. The ranges of variation for stroke patients are not similar to the healthy subjects. As the patients have different levels of impairment, the flexion of each finger has higher ranges across stroke patients with several outliers, except in the case of the ring finger and little finger where the variation was quite small (max 45 degrees). This might be due to the limited mobility of these fingers and therefore the patients had to rely on the thumb, index finger, and middle finger to grasp the objects.

**Figure 6:**
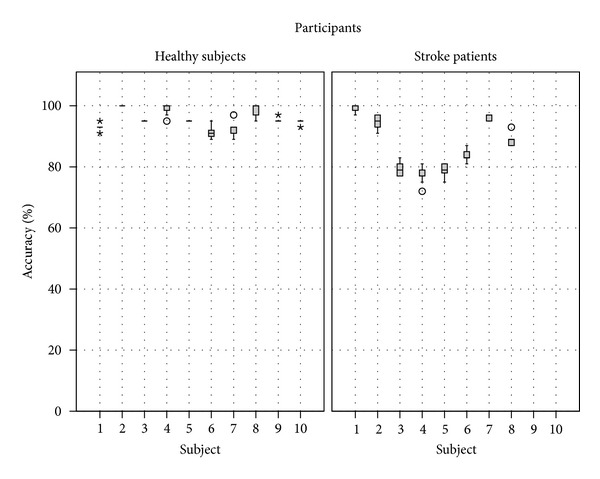
Results of the accuracy of gesture recognition per participant. The left graph presents the results for healthy participants and the right one for stroke patients.

The results of the accuracies of recognition of the different gestures per participant are presented in Figure 6. The variation of accuracies of healthy subjects (between 89 and 100%) was smaller than the ones of stroke patients (between 72 and 100%).

**Figure 7:**
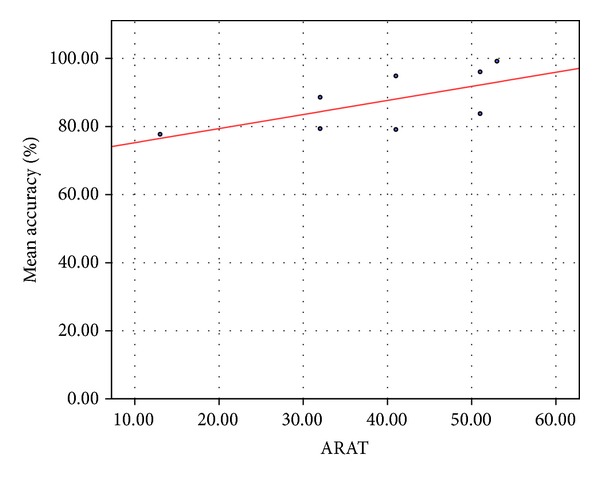
Association between accuracy of recognition and the results of the ARAT.

The correlation results of the recognition accuracies with the results of the arm motor recovery tests are presented in Table 5. The higher positive correlations are between the accuracies and the ARAT test, especially to sections A (grasp subtest) and C (pinch subtest). This is not surprising given that the ARAT specifically assesses dexterity; while FM is a much broader measure of motor impairment.  Figure 7 presents accuracy and ARAT score values for each subject to visually show the high association.

### 3.2. Grasp Evaluation Results

**Figure 8:**
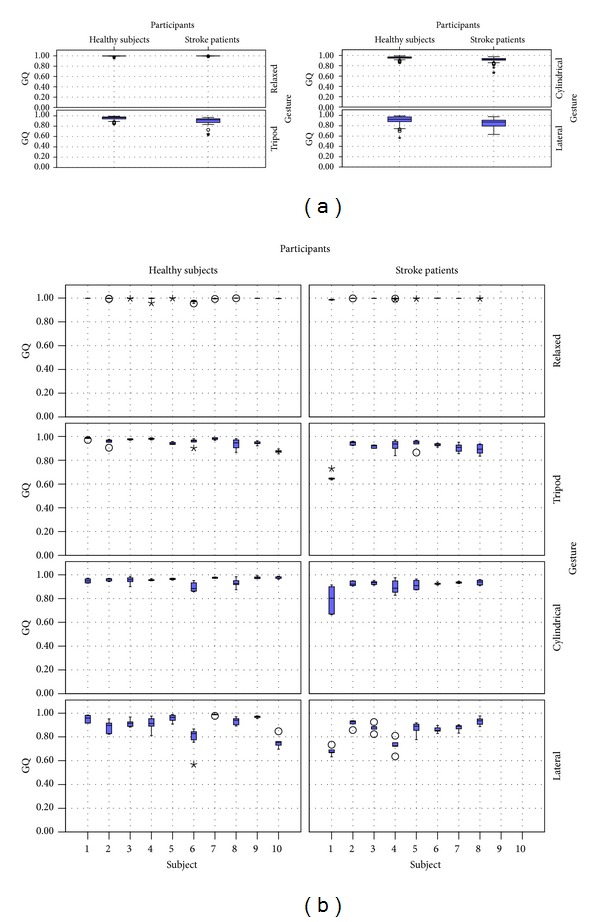
Results of the grasp quality (GQ) for healthy subjects and stroke patients per gesture: (a) summary of the results and (b) results showing variability per participant.

The results of the evaluation of each of the grasps using the proposed quality measure GQ are presented in Figure 8. The difference between the participant's type and the grasp performed are shown in Figure 8(a). The healthy subjects presented smaller variations than the stroke patients and their median grasp quality was higher. The higher variation was presented while the participants performed the lateral grasp: the grasp quality varied 24% (0.75–0.99) for healthy patients and 28% (0.67–0.95) for stroke patients.

The variability between different participants is shown in Figure 8(b). The grasp performed by healthy participants got a measure of quality above 0.85 for the relaxed, tripod, and cylindrical grasps. However, performing the lateral grasp presented a higher variability, especially for subjects 6 and 10 who obtained grasp qualities as low as 0.57 and 0.73, respectively. Stroke patients presented a higher variability between subjects, but in general there was a consistent grasp quality per subject and gesture (maximum 17%)—except patient 1 performing the cylindrical grasp who showed a variation of 25% (0.66–0.91).

The correlation results of the grasp quality with the results of the arm motor recovery tests are presented in Table 6. In general, there are no significant correlations between the tests and the results obtained from the quality measure, except for a negative correlation with the Fugl-Meyer coordination test performing the relax posture and the tripod grasp.

In order to assess how the quality measure GQ was influenced by the type of participant (healthy subject versus stroke patient), the gesture and the specific subject performing the grasps, a multiple regression analysis was performed. The results of the analysis are presented in Table 7. The *R* value of 0.78 shows that the predictors are a good estimation of the quality measure. The *R* ^2^ indicates that the predictors account for 60.9% of the variation of the grasp quality measure and as the adjusted *R* ^2^ is similar to it, it means that the model is able to generalize. Also, the standard error 0.04365 indicates that most sample means from the estimated values are similar to the quality measure means. The *F* change is an important value, as it indicates that this model causes *R* ^2^ to change from zero to 0.609, which is significant with a probability less than 0.001.

## 4. Discussion

In this study, we examined the performance of a technique based on support vector machines for the recognition of hand gestures using the finger flexion/extension angles, comparing a group of healthy subjects with a group of poststroke patients. The results for stroke patients in general show lower accuracies, lower true positive rates, and greater false positive rates than the ones performed by the healthy subjects. This is due to the fact that patient's impairment after stroke affects their ability to reproduce gestures with small variations and in a repeatable way. However, we hypothesized that the accuracy for gesture recognition for stroke patients can provide an insight into patients' ability to consistently reproduce gestures. This was corroborated with the high correlation between the recognition accuracies and the scores of the ARAT (*C* = 0.651 for ARAT total) showing that the accuracy of recognition can be used as a measure of the grasping capabilities for the impaired hand. Therefore, this method shows potency to be used to detect changes related to lack of ability to reproduce gestures in a consistent way.

The results of the grasp quality measure showed that the healthy subjects presented smaller variations than the stroke patients and their grasp quality is higher showing that the measure can be used to assess the capabilities of stroke patients to perform grasp postures in a similar way than healthy people. However, the variation between healthy subjects is higher than we expected. This can be due to the mechanical design of the orthosis, as the readings of the sensors could vary from orthosis to orthosis depending on the level of tension of the elastic cords. With a more accurate device, as the currently developed next version of the SCRIPT orthosis, it is expected that the variability of the healthy postures will be reduced which will give more consistent high quality values for the healthy participants and therefore would be more reliable for the evaluation of stroke patient grasps. Also, a broader study with a larger number of healthy subjects and stroke patients could give a more accurate measure of the quality of the grasp.

Also, the lack of correlation between the grasp quality GQ and the results of the arm motor recovery tests could indicate that this measure is perhaps providing different information about the patients, indicating specifically their level of ability to perform each of the different gestures which is not specifically measured by the common tests. The results of the multiple regression analysis showed that the type of participant (healthy subject or stroke patient), the grasp and the specific subject can account for 61% of the grasp quality variability, which indicate presence of other indicators that have not been considered in our model. We hypothesize that these variations could be due to differences influenced by gender, the hand performing the gestures (dominant or nondominant), or the level of disability of the stroke patients. Future work is needed to study the influences of these factors when more participants and more accurate readings are available.

The technique presented in this paper will be used for the recognition of hand postures needed for the activities of daily life while patients playing rehabilitation games with the SCRIPT orthosis over a period of six weeks. With these results, more exhaustive analysis can be performed which could provide insights into improvements on the ability to perform the grasp postures over time influencing the overall motor performance.

## 5. Conclusion

The results obtained with this study show that a technique based on support vector machines can be used to recognize different grasp gestures for stroke patients with a valid accuracy while playing rehabilitation games wearing a specially designed exoskeleton for their rehabilitation. This will allow the training of various grasp postures to improve the performance of these postures needed for several activities of daily living. Moreover, we showed that the accuracy of recognition can be used to assess the ability of the stroke patients to consistently repeat the gestures and the proposed grasp quality GQ can be used to measure the capabilities of the stroke patients to perform grasp postures in a similar way than healthy people. These two measures could be used as complementary measures to the other upper limb motion tests such as ARAT and they can be potentially applied to evaluate the grasp performance of patients using other orthosis able to measure finger flexion.

## Figures and Tables

**Table 1:** Details of healthy participants.

Id	Country	Age	Gender	Dominant
1	UK	27	Male	Right
2	UK	32	Male	Right
3	UK	33	Male	Right
4	UK	30	Female	Right
5	UK	32	Female	Right
6	Italy	47	Female	Left
7	Italy	50	Male	Right
8	Italy	52	Female	Right
9	Italy	50	Male	Left
10	Italy	51	Female	Left

**Table 2:** Details of the stroke patients.

Id	Country	Age (yrs.)	Time since acute event	Gender	Affected	Dominant	Stroke type	ARAT (max 57 points)					FM (max 66 points)			
								A	B	C	D	Total	Proximal	Distal	Coordination	Total
1	Holland	69	13 months	Male	Left	Left	Ischemia	18	10	17	8	53	29	16	4	49
2	Italy	73	7 months	Female	Left	Right	Ischemia	12	8	12	9	41	28	12	3	43
3	Italy	88	6 months	Male	Left	Right	Ischemia	12	8	6	6	32	27	12	4	43
4	Italy	83	7 months	Male	Left	Right	Ischemia	6	4	0	3	13	28	11	3	42
5	Italy	85	6 months	Male	Right	Right	Ischemia	12	8	12	9	41	28	12	3	43
6	Italy	72	6 months	Male	Left	Right	Ischemia	18	12	12	9	51	28	23	3	54
7	Italy	81	7 months	Female	Left	Right	Ischemia	18	12	12	9	51	35	24	3	62
8	Italy	69	7 months	Male	Left	Right	Ischemia	12	8	6	6	32	26	12	3	41

ARAT = arm scores of the action research arm test; FM = arm scores of the Fugl-Meyer motor assessment.

**Table 3:** Training time and true positive rate (%) between the training and testing phases.

		Mean training time (ms)	Mean training TPR (%)	Mean testing TPR (%)
Healthy subjects	1	1.800 ± 0.6	58 ± 14	87 ± 2
	2	1.733 ± 0.6	100 ± 0	100 ± 0
	3	2.200 ± 0.7	84 ± 14	91 ± 0
	4	1.933 ± 0.5	79 ± 25	98 ± 3
	5	1.733 ± 0.6	84 ± 14	91 ± 0
	6	1.867 ± 0.5	58 ± 10	83 ± 4
	7	2.200 ± 1.4	63 ± 9	85 ± 4
	8	2.267 ± 1.6	98 ± 8	97 ± 4
	9	2.200 ± 0.6	31 ± 9	91 ± 1
	10	1.867 ± 0.5	84 ± 16	90 ± 2

		1.980 ± 0.5	74 ± 24	91 ± 6

Stroke patients	1	2.600 ± 0.7	100 ± 0	99 ± 2
	2	2.200 ± 0.4	61 ± 15	91 ± 4
	3	2.200 ± 0.4	38 ± 6	59 ± 4
	4	1.933 ± 0.7	38 ± 11	56 ± 4
	5	2.467 ± 0.6	38 ± 6	58 ± 4
	6	2.067 ± 0.6	33 ± 6	68 ± 5
	7	2.067 ± 0.3	47 ± 19	93 ± 2
	8	2.133 ± 0.4	43 ± 26	78 ± 3

		2.208 ± 0.6	50 ± 25	75 ± 17

**Table 4:** Correlation between performance measures.

Performance measures	Participant	Pearson correlation
Accuracy versus TPR	Healthy subject	0.999∗
	Stroke patient	1.000∗

Accuracy versus FPR	Healthy subject	−0.973∗
	Stroke patient	−0.997∗

*Correlation is significant at the 0.01 level (2-tailed).

**Table 5:** Correlation between accuracy and common arm motor recovery tests.

Test compared with mean accuracy	Pearson correlation	Sig.
ARAT_total	0.651	0.040
ARAT_A	0.630	0.047
ARAT_B	0.532	0.087
ARAT_C	0.680	0.032
ARAT_D	0.502	0.103
FM_total	0.461	0.125
FM_proximal	0.481	0.114
FM_distal	0.386	0.172
FM_coordination	0.141	0.370

**Table 6:** Correlation between the grasp quality measure (GQ) and common arm motor recovery tests.

Test compared with GQ	Relax posture		Tripod grasp		Cylindrical grasp		Lateral grasp	
	Pearson correlation	Sig.	Pearson correlation	Sig.	Pearson correlation	Sig.	Pearson correlation	Sig.
ARAT_total	−0.365	0.187	−0.387	0.172	−0.247	0.278	0.078	0.427
ARAT_A	−0.375	0.180	−0.443	0.136	−0.247	0.278	0.008	0.492
ARAT_B	−0.139	0.371	−0.214	0.305	−0.007	0.494	0.209	0.310
ARAT_C	−0.527	0.090	−0.502	0.102	−0.434	0.141	−0.055	0.448
ARAT_D	−0.077	0.429	−0.051	0.452	0.020	0.482	0.347	0.200
FM_total	−0.117	0.391	−0.133	0.376	−0.005	0.496	−0.037	0.465
FM_proximal	0.130	0.380	−0.083	0.422	−0.002	0.498	−0.071	0.434
FM_distal	−0.042	0.461	−0.086	0.419	0.044	0.459	0.023	0.479
FM_coordination	−0.638∗	0.044	−0.660∗	0.038	−0.574	0.069	−0.441	0.137

*Correlation is significant at the 0.05 level (1-tailed).

ARAT = arm scores of the action research arm test; FM = arm scores of the Fugl-Meyer motor assessment.

**Table 7:** Multiple regression results.

Model	*R*	*R* square	Adjusted *R* square	Std. Error of the Estimate	Change statistics				
					*R* Square change	*F* change	df1	df2	Sig. *F* change
1	0.780	0.609	0.597	0.04365	0.609	53.469	20	687	0.000
